# Does the Addition of Cetuximab to Radiochemotherapy Improve Outcome of Patients with Locally Advanced Rectal Cancer? Long-Term Results from Phase II Trials

**DOI:** 10.1155/2015/273489

**Published:** 2015-03-15

**Authors:** M. Kripp, K. Horisberger, S. Mai, P. Kienle, T. Gaiser, S. Post, F. Wenz, K. Merx, R.-D. Hofheinz

**Affiliations:** ^1^III. Medizinische Klinik, Universitätsmedizin Mannheim, Universität Heidelberg, Theodor-Kutzer-Ufer 1-3, 68167 Mannheim, Germany; ^2^Chirurgische Klinik, Universitätsmedizin Mannheim, Universität Heidelberg, Theodor-Kutzer-Ufer 1-3, 68167 Mannheim, Germany; ^3^Klinik für Strahlentherapie und Radioonkologie, Universitätsmedizin Mannheim, Universität Heidelberg, Theodor-Kutzer-Ufer 1-3, 68167 Mannheim, Germany; ^4^Pathologisches Institut, Universitätsmedizin Mannheim, Universität Heidelberg, Theodor-Kutzer-Ufer 1-3, 68167 Mannheim, Germany; ^5^Interdisziplinäres Tumorzentrum, Universitätsmedizin Mannheim, Universität Heidelberg, Theodor-Kutzer-Ufer 1-3, 68167 Mannheim, Germany

## Abstract

*Purpose*. The addition of cetuximab to radiochemotherapy (RCT) failed to improve complete response rates in locally advanced rectal cancer (LARC). We report the long-term results in patients treated within two sequential clinical trials. *Methods*. Patients receiving neoadjuvant RCT using capecitabine and irinotecan (CapIri) within a phase I/II trial or CapIri + cetuximab within a phase II trial were evaluated for analysis of disease-free survival (DFS) and overall survival (OS). KRAS exon 2 mutational status had been analyzed in patients receiving cetuximab. *Results*. 37 patients from the CapIri trial and 49 patients from the CapIri-cetuximab treatment group were evaluable. Median follow-up time was 75.2 months. The 5-year DFS rate was 82% (CapIri) and 79% (CapIri-cetuximab) (*P* = 0.62). The median OS was 127.4 months. 5-year OS was 73% for both groups (CapIri and CapIri-cetuximab) (*P* = 0.61). No significant difference in DFS (*P* = 0.86) or OS (*P* = 0.39) was noticed between patients receiving CapIri and those receiving CapIri-cetuximab with KRAS wild-type tumors. *Conclusions*. As the addition of cetuximab did not improve neither DFS nor OS it should not play a role in the perioperative treatment of patients with LARC, not even of patients with (K)RAS WT tumors.

## 1. Introduction

Neoadjuvant radiochemotherapy (RCT) with fluoropyrimidines followed by total mesorectal excision (TME) is a standard therapy for locally advanced rectal cancer (LARC) [1]. Epidermal growth factor receptor (EGFR) is overexpressed in 50–70% of primary rectal cancers [2] and is related to decreased pathological complete response (pCR), disease-free survival (DFS), and overall survival (OS) [3, 4]. Thus, several studies sought to investigate the efficacy of combined treatment regimens using targeted agents directed against EGFR in conjunction with RCT [5–11].

Cetuximab is a chimeric anti-EGFR monoclonal antibody approved for the treatment of metastatic colorectal cancer. In patients with metastatic colorectal cancer it has been demonstrated that the benefit is limited to patients with wild-type (WT) (K)RAS tumors [12, 13]. However, the addition of anti-EGFR antibodies to RCT failed to improve pathological complete response rates in patients with LARC [6–10, 14–18]. Only few randomized trials have been reported so far. EXPERT-C, a multicenter randomized phase II trial, investigated the addition of cetuximab to preoperative induction chemotherapy and RCT in 165 patients with high-risk rectal cancer. In 149 patients with KRAS/BRAF WT tumors the addition of cetuximab to capecitabine and oxaliplatin (CapOx) led to a significant increase in radiologic response rates but the primary endpoint (increasing pCR rate) was missed. Regarding overall survival, a significant benefit for patients in the cetuximab group was noticed in the initial study report [11]. However, after a median follow-up of 63.8 months the improvement of overall survival in RAS WT patients was still clinically meaningful but did not retain statistical significance (5-year overall survival CapOx-Cetuximab 83.8% and CapOx 70.0%; *P* = 0.20) [19].

Another randomized phase II trial evaluated the addition of the fully human anti-EGFR antibody panitumumab to a capecitabine-based RCT regimen as neoadjuvant treatment for KRAS WT LARC in 68 patients staged cT3/4 or N+. The primary endpoint was the rate of pathological near-complete or complete remissions applying Dworak regression grading [20]. While the pCR rate was not increased by the addition of panitumumab, the rate of near-complete plus complete remissions was substantially higher (53% versus 32%). Long-term data have not been reported [21].

In view of the paucity of randomized studies and few follow-up data, we herein report a long-term analysis of study patients receiving either capecitabine/irinotecan (CapIri) or CapIri-cetuximab based RCT for LARC within two sequential clinical trials using comparable inclusion criteria.

## 2. Material and Methods

### 2.1. Patients

In the current analysis we evaluated long-term results of patients receiving neoadjuvant RCT using CapIri (within a phase I/II trial) or CapIri + cetuximab (within a phase II trial). These trials were conducted on a monocentric basis at the Departments of Oncology, Radiotherapy and Radiooncology, and Surgery at the University Hospital of Mannheim, University of Heidelberg, Germany. The trials have been reported in detail previously [9, 22, 23]. Briefly, patients with LARC were scheduled to receive CapIri (i.e., irinotecan 50 mg/m² on days 1, 8, 15, 22, and 29 in combination with capecitabine 500–625 mg/m² b.i.d. on days 1 through 38) or CapIri-cetuximab (cetuximab 400 mg/m² on day 1 and 250 mg/m² on days 8, 15, 22, and 29 in combination with irinotecan 40 mg/m² on days 1, 8, 15, 22, and 29 and capecitabine 500 mg/m² b.i.d. on days 1 through 38). Patients included in both trials with local tumor relapse or metastases (even if deemed resectable) were excluded for the present analysis. Surgery was scheduled to take place 4−6 weeks after termination of RCT.

All patients included in the current analysis had histologically confirmed rectal cancer staged with endoscopic ultrasound as cT3-T4 or N+ tumors. MRI was not used as an inclusion criterion. Distant metastases had been excluded with CT scan. Further patient eligibility criteria comprised Eastern Cooperative Oncology Group performance status ≤ 2, age ≥ 18 years, adequate bone marrow (leukocyte count > 3000/*μ*L and platelet count > 100,000/*μ*L), and sufficient renal (serum creatinine ≤ 1.4 mg/dL or creatinine clearance > 60 mL/min) and hepatic (bilirubin ≤ 2 mg/dL) function. Patients were excluded if they had other forms of cancer or had known hypersensitivity to 5-FU (5-fluorouracil), irinotecan, or cetuximab.

All studies were conducted according to the Declaration of Helsinki. The protocols were approved by the local ethical committee. Written informed consent was obtained from each patient before study entry.

Resection specimens were pathologically analyzed and pCR was defined as complete absence of tumor cells.

KRAS exon 2 mutational status had been analyzed in patients having received CapIri and cetuximab using formalin-fixed, paraffin-embedded tumor tissue obtained prior to the start of RCT [24]. Briefly, for analysis of KRAS mutations, microdissection of tumor tissue was carried out, and DNA (deoxyribonucleic acid) was subjected to (semi)nested PCR (polymerase chain reaction) amplification of exon 2 of the KRAS gene containing codons 12 and 13. Further mutation analyses (KRAS exons 3 and 4 and NRAS exons 2–4) were not performed because written conformed consent had been given only for KRAS exon 2 status.

### 2.2. Statistical Analyses

Disease-free survival was defined as time to local recurrence, metastases, or death whichever occurred first, and overall survival was calculated as the time from start of treatment until death. Time-to-event data were calculated using the Kaplan-Meier method. Comparisons between the groups were performed using the log-rank (Mantel-Cox) test.

To compare clinical and pathological parameters, including age, gender, T/N level downstaging, and pCR rates we used the unpaired *t*-test, Fisher's exact test, and Chi square, respectively. A 2-sided *P* value of *P* ≤ 0.05 was considered significant.

Statistical analyses and figures were performed with GraphPad Prism 5.

## 3. Results

### 3.1. Patients' and Tumor Characteristics

A total of 93 patients were included in both trials between May 2002 and February 2008: *n* = 43 patients were treated with CapIri and *n* = 50 patients with CapIri and cetuximab. A total of *n* = 7 patients had to be excluded for the current analysis because they had been treated within these trials with distant metastases or a local relapse (*n* = 6 were excluded from the CapIri cohort and *n* = 1 from the CapIri-cetuximab cohort, resp.). In summary, *n* = 37 patients from the CapIri trial and *n* = 49 patients from the CapIri-cetuximab treatment group were evaluable for the current analysis.

Both groups did not differ from each other regarding gender, age, clinical T and N, and UICC (Union internationale contre le cancer) status. Patients' and tumor characteristics are depicted in Table 1.

In 45 out of 49 patients treated with CapIri and cetuximab analysis of KRAS mutations could be done. Of these, 32 patients (71.1%) were KRAS WT, and 13 patients (28.9%) had tumors harboring KRAS mutations.

### 3.2. Pathohistological Analysis of Resection Specimen

A total of 11 patients achieved a pCR, seven (19%) with CapIri and four (8%) with CapIri-cetuximab. The difference was not significant (n.s.).

A T-downstaging (defined as ypT0-2, N0) occurred in 30 patients (24% with CapIri and 43% with CapIri-cetuximab; n.s.). A nodal negative tumor upon resection occurred in 59 patients (62% with CapIri and 73% with CapIri-cetuximab; n.s.). Data are shown in Table 2.

### 3.3. Disease-Free and Overall Survival

All patients were followed up for survival time; no patient was lost to follow-up.

At the time of analysis, a total of 30 patients had died (34.9%). Median follow-up time for all patients was 75.2 months. Follow-up data are shown in Table 3. Two out of 37 patients treated with CapIri had local recurrence, four had metastatic disease, and two patients had both, local recurrence and metastatic disease. Thus, a total of 10.8% of patients had local recurrence and 16.2% had distant metastases, adding to an overall recurrence rate of 21.6%. Within the CapIri-cetuximab group the results were as follows: 10 out of 49 patients had distant failure (20.4%), and one patient had a local relapse (2.0%) adding to an overall recurrence rate of 22.4%.

**Figure 1:**
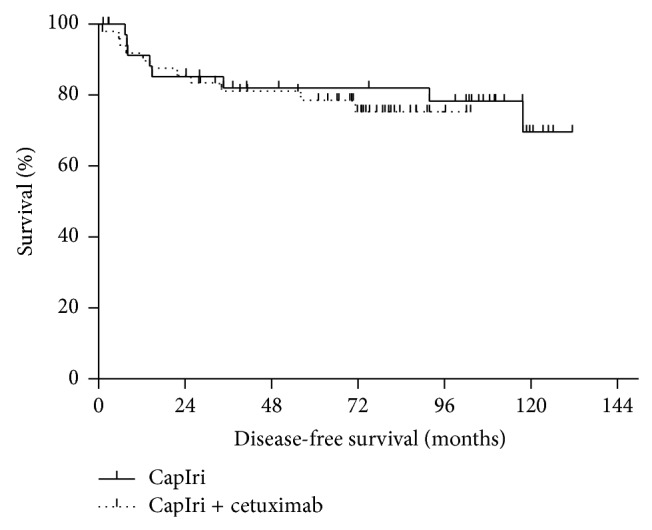
Disease-free survival of patients with locally advanced rectal cancer receiving capecitabine/irinotecan +/− cetuximab based chemoradiotherapy as preoperative treatment (*n* = 86). Shown are survival curves of patients treated with CapIri (solid curve; *n* = 37) versus patients treated with CapIri + cetuximab (dotted curve; *n* = 49), *P* = 0.62.

Taken together, the 5-year DFS rate was 80% (82% for CapIri patients and 79% for CapIri-cetuximab patients; *P* = 0.62; Figure 1). The analysis of the cetuximab group according to KRAS status revealed a 5-year DFS of 84% for patients with KRAS WT and 74% for patients with KRAS mutation. This difference was not statistically significant (*P* = 0.71). Similarly, no significant difference in DFS was noticed between CapIri patients and patients with KRAS WT tumors receiving CapIri-cetuximab (*P* = 0.86).

**Figure 2:**
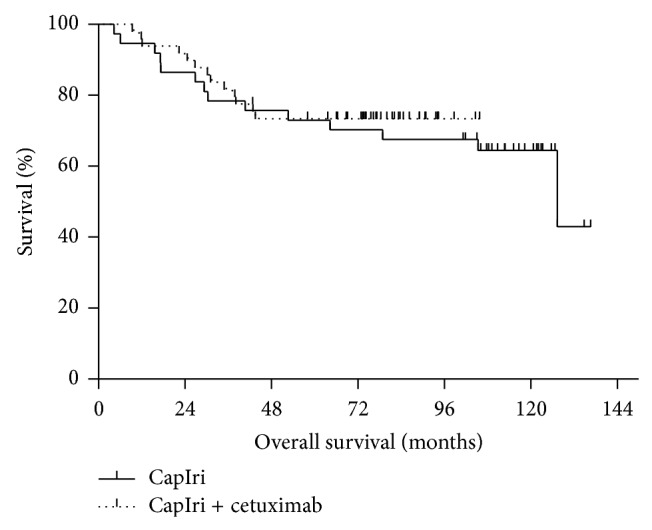
Overall survival of patients with locally advanced rectal cancer receiving capecitabine/irinotecan +/− cetuximab based chemoradiotherapy as preoperative treatment (*n* = 86). Shown are survival curves of patients treated with CapIri (solid curve; *n* = 37) versus patients with KRAS WT receiving CapIri + cetuximab (dotted curve; *n* = 49), *P* = 0.61.

The 5-year overall survival rate was 73%. The median overall survival for all patients was 127.4 months. No significant difference was seen between patients receiving CapIri or CapIri-cetuximab: 5-year overall survival was 73% for both groups (*P* = 0.61, Figure 2). A numerical difference was found regarding 5-year overall survival in patients treated with CapIri-cetuximab with respect to the KRAS mutational status: 5-year overall survival rate was 78% for patients with KRAS WT tumors but only 62% for patients with tumors harboring KRAS mutations. However, no statistical significance was achieved (*P* = 0.24).

**Figure 3:**
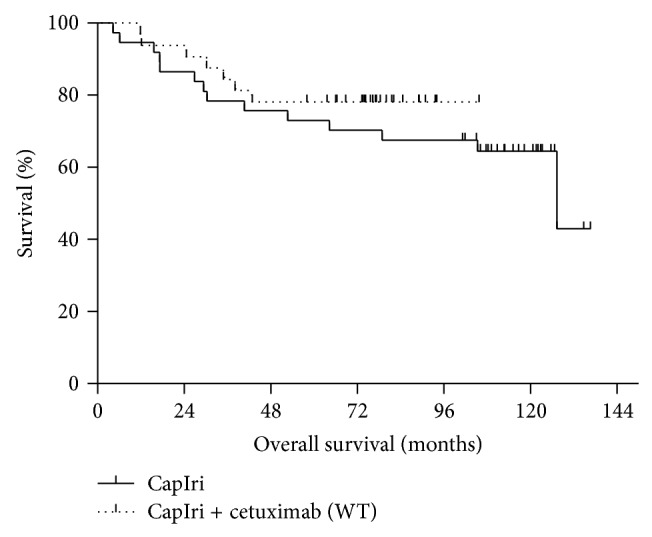
Overall survival of patients with locally advanced rectal cancer receiving capecitabine/irinotecan +/− cetuximab based chemoradiotherapy as preoperative treatment (*n* = 86). Shown are survival curves of patients treated with CapIri (solid curve; *n* = 49) versus patients with KRAS WT receiving CapIri + cetuximab (dotted curve; *n* = 32), *P* = 0.39.

Similarly, no statistical significant difference between patients receiving CapIri and those receiving CapIri-cetuximab with KRAS WT tumors was noticed (*P* = 0.39; Figure 3).

## 4. Discussion

Neoadjuvant RCT or short-course radiotherapy followed by TME is a standard treatment for LARC [25, 26]. Although the local recurrence rate is generally below 10%, systemic recurrence still occurs in about 25–30% of cases [25, 26]. Thus far, alternative strategies using more intensive chemotherapeutic regimens and/or combination treatments with targeted agents failed to demonstrate a significant advantage over standard RCT [5–10, 14–17, 27–29]. Particularly, early efficacy endpoints, such as the pCR rate, could not be improved by addition of an anti-EGFR antibody to neoadjuvant RCT with capecitabine/5-FU alone or in combination with irinotecan or oxaliplatin [6–10, 14–17, 21].

Undoubtedly, patients achieving a pCR have an excellent long-term prognosis [30]. However, there is an ongoing debate if pCR may be used as a valid surrogate endpoint for rectal cancer trials [31]. In this regard, the results of EXPERT-C are of interest. A multicenter randomized phase II trial compared neoadjuvant oxaliplatin, capecitabine (CapOx), and preoperative radiotherapy with or without cetuximab in patients with high-risk LARC [11]. The trial was originally designed to detect a 20% improvement in pCR. The protocol was amended to the primary endpoint of complete response (pCR or radiologic complete response) in patients with KRAS/BRAF wild-type tumors. However, the primary endpoint “improved pathological complete response rate” or “radiologic complete response rate in patients with KRAS/BRAF WT tumors” was not met. The addition of cetuximab resulted in a significant increase in radiologic response and a clinically meaningful benefit in overall survival [11]. After longer follow-up, the difference remained still clinically significant with a 12% difference for 5-year overall survival (84.3% versus 72.3%). However, statistical significance could not be demonstrated anymore [19]. Likewise, the 5-year DFS was not statistically significantly different (75.4% versus 67.8%; *P* = 0.23).

The herein presented analysis is the first comparative data of long-term results of European patients with LARC receiving intensive neoadjuvant RCT with capecitabine and irinotecan with or without cetuximab within two sequential clinical trials. Although not randomized the patient population treated within these trials were well balanced, and inclusion criteria and staging measures for both studies were similar. Moreover, the patients were treated by the same multidisciplinary team within identical circumstances. Our findings indicate, comparable to the EXPERT-C experience, that the addition of cetuximab did not improve neither 5-year DFS nor OS. We observed only a small numerical 5-year survival benefit of 5% in favor of the KRAS WT patients receiving cetuximab compared to the (KRAS WT and mutated) CapIri patients (78% versus 73%; *P* = 0.41). Comparable to our results the analysis of two Korean trials using similar RCT regimens did not reveal a difference within the KRAS WT group [31]. The 5-year DFS rate in our patient population was virtually identical between the CapIri and the CapIri-cetuximab KRAS WT patients (84% and 82%, resp.). Again, the same observation has been described in the Korean data analysis [32].

It is noteworthy that in our series pCR rates were rather low within the cetuximab group by analogy with what has been observed in other trials like in the abovementioned EXPERT-C study. However, it is interesting that the rate of patients with ypT0-2 N0 tumors was higher in the cetuximab group (43% versus 24%), and the number of patients with pathological nodal negative disease was also higher in the cetuximab group (73% versus 62%) compared to the patients treated without cetuximab. It could be argued that cetuximab treated patients should undergo surgery after a longer period after completion of RCT. Indeed, range of time to resection was wider than planned in general but smaller in the cetuximab group compared to the patients treated without cetuximab. Moreover, the higher rate of cT2 tumors in favor of lower rates of cT3 and cT4 tumors in the cetuximab group could be a possible bias for the higher rate of ypT0-2 N0 downstaging in the cetuximab group. Someone may also speculate if tumor remission “beyond pCR” is something that is overlooked when focusing on pCR rate only. A similar observation has been made in the SAKK study which investigated the addition of panitumumab to a capecitabine-based RCT [21]. Using the regression grading described by Dworak and coworkers pCR rate was not increased by the addition of panitumumab. However, the rate of near-complete plus complete remissions was clearly higher (53% versus 32%). In this regard, it is noteworthy that the rate of local recurrences was rather small within the cetuximab group in our trial (one out of 49 patients after a follow-up of 72 months), while the rate of local recurrences within the chemotherapy without cetuximab group was higher (4 out of 37 patients after a follow-up of 105 months). Likewise, in the EXPERT-C trial the local recurrence rate in KRAS WT patients was numerically lower as well in the cetuximab group (5.4% versus 2.4% local relapse; hazard ratio 0.46, n.s.).

However, despite some hints of potential activity of cetuximab in the perioperative treatment of patients with LARC (i.e., numerical better survival rates, lower rates of local relapse, and indications for improved primary tumor shrinkage “beyond pCR”) the data at hand is not convincing so far. As recently reported, a benefit could not be demonstrated in RAS WT patients in the EXPERT-C trial. The explanation for the lack of activity is unclear. Among other arguments it has been speculated that cetuximab if delivered concurrently with radiation could potentially abolish additive effects of 5-FU, by inhibiting proliferation. Moreover preclinical data suggests that the sequencing of chemotherapy, EGFR inhibition, and radiation may be clinically significant. Additionally, it is known that cetuximab should not be combined with capecitabine in patients with metastatic colorectal cancer [33]. However, unfortunately most of the RCT/cetuximab trials in patients with LARC have been conducted using capecitabine.

In view of several negative studies with cetuximab based RCT, it seems unlikely that a definitive randomized phase III trial will be undertaken in LARC. However, lately it could be shown in the EXPERT-C study population that in patients with a TP 53 WT tumor the use of cetuximab in conjunction with RCT significantly improved the 5-year overall and disease-free survival (e.g., HR overall survival 0.16, 95% CI 0.04–0.70, and *P* = 0.02). While TP53 status was not of prognostic value it emerged as a predictive factor for cetuximab benefit. The benefit from cetuximab in patients with TP53 wild-type tumors was independent of RAS [34].

Finally, there is currently no indication that anti-EGFR monoclonal antibodies, namely, cetuximab, should play a role in the perioperative treatment of all patients with locally advanced rectal cancer, not even of patients with (K)RAS WT tumors. Nevertheless, the recent findings that the TP53 WT status may serve as a strong predictor for cetuximab efficacy in the RCT of LARC may stimulate further retrospective and maybe prospective studies to elucidate treatment effects in smaller subgroups of patients.

## Figures and Tables

**Table 1:** Characteristics of patients with locally advanced rectal cancer receiving capecitabine/irinotecan +/− cetuximab based chemoradiotherapy as preoperative treatment.

	Capecitabine/irinotecan (*n* = 37)	Capecitabine/irinotecan + cetuximab (*n* = 49)	*P* value
Gender, *n* (%)			
Male	29 (78)	33 (67)	
Female	8 (22)	16 (33)	
Fisher's exact test			0.33

Age (years); median (range)	60 (34–82)	57 (33–80)	
Unpaired *t*-test			0.79

Clinical TNM stage, *n* (%)			
cT1	0 (0)	0 (0)	
cT2	4 (11)	9 (18)	
cT3	28 (76)	36 (73)	
cT4	4 (11)	4 (8)	
cT*x *	1 (3)	0 (0)	
Chi square			0.62
cN−	6 (16)	15 (31)	
cN+	31 (84)	34 (69)	
Fisher's exact test			0.14

Tumor distance from anal verge (cm); median (range)	6 (2–13)	7 (0–13)	
Unpaired *t*-test			0.77

KRAS status, *n* (%)			
KRAS wild-type	n.a.	32 (65)	
KRAS mutation	n.a.	13 (27)	
KRAS unknown	n.a.	4 (8)	

Surgical technique			
Low anterior resection	31	41	
Abdominoperineal resection	6	7	
Hartmann's operation	0	1	
Chi square			0.67

Time to resection (days), median (range)	39 (21–79)	38 (21–67)	
Unpaired *t*-test			0.94

n.a.: not applicable.

**Table 2:** Pathohistological results of patients with locally advanced rectal cancer receiving capecitabine/irinotecan +/− cetuximab based chemoradiotherapy as preoperative treatment.

	Capecitabine/irinotecan (*n* = 37)	Capecitabine/irinotecan + cetuximab (*n* = 49)	*P* value
ypT downstaging, *n* (%)			
ypT0	7 (19)	4 (8)	
ypT1	5 (14)	1 (2)	
ypT2	11 (30)	18 (37)	
ypT3	12 (32)	24 (49)	
ypT4	2 (5)	2 (4)	
Chi square			0.11
ypN−	23 (62)	36 (73)	
ypN+	14 (38)	13 (27)	
Fisher's exact test			0.35

R0 resection, *n* (%)			
	36 (97)	48 (98)	
Fisher's exact test			1.00

Pathologic complete remission ypT0 pN0, *n* (%)			
	7 (19)	4 (8)	
Fisher's exact test			0.19

T-downstaging (ypT0-2, pN0), *n* (%)			
	9 (24)	21 (43)	
Fisher's exact test			0.11

**Table 3:** Follow-up of patients with locally advanced rectal cancer receiving capecitabine/irinotecan +/− cetuximab based chemoradiotherapy as preoperative treatment (*n* = 98).

	Capecitabine/irinotecan *n* = 37	Capecitabine/irinotecan + cetuximab *n* = 49
Alive	23	36
Dead	14	13
Follow-up (months); median (range)	105.5 (1.3–133.7)	71.9 (7–103.3)
